# Current Situation of Palytoxins and Cyclic Imines in Asia-Pacific Countries: Causative Phytoplankton Species and Seafood Poisoning

**DOI:** 10.3390/ijerph19084921

**Published:** 2022-04-18

**Authors:** Young-Sang Kim, Hyun-Joo An, Jaeseong Kim, You-Jin Jeon

**Affiliations:** 1 Laboratory of Marine Bioresource Technology, Department of Marine Life Science, School of Marine Biomedical Sciences, Jeju National University, Jeju City 63243, Korea; medieval032@gmail.com; 2 Marine Science Institute, Jeju National University, Jeju City 63333, Korea; 3 Asia Glycomics Reference Site, Chungnam National University, Daejeon 34134, Korea; hjan@cnu.ac.kr; 4 Graduate School of Analytical Science and Technology, Chungnam National University, Daejeon 34134, Korea; 5 Water and Eco-Bio Corporation, Kunsan National University, Kunsan 54150, Korea; kimjs90@kunsan.ac.kr

**Keywords:** marine biotoxin, palytoxin, cyclic imine, harmful algal bloom

## Abstract

Among marine biotoxins, palytoxins (PlTXs) and cyclic imines (CIs), including spirolides, pinnatoxins, pteriatoxins, and gymnodimines, are not managed in many countries, such as the USA, European nations, and South Korea, because there are not enough poisoning cases or data for the limits on these biotoxins. In this article, we review unregulated marine biotoxins (e.g., PlTXs and CIs), their toxicity, causative phytoplankton species, and toxin extraction and detection protocols. Due to global warming, the habitat of the causative phytoplankton has expanded to the Asia-Pacific region. When ingested by humans, shellfish that accumulated toxins can cause various symptoms (muscle pain or diarrhea) and even death. There are no systematic reports on the occurrence of these toxins; however, it is important to continuously monitor causative phytoplankton and poisoning of accumulating shellfish by PlTXs and CI toxins because of the high risk of toxicity in human consumers.

## 1. Introduction

### 1.1. Harmful Algal Blooms (HABs) and Shellfish Poisoning

**Figure 1:**
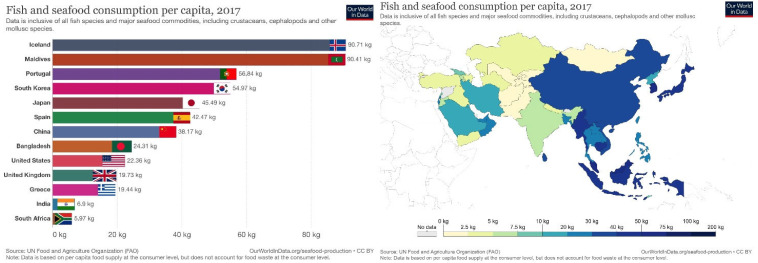
Fish and seafood consumption per capita, 2017. Data based on per capita food supply at the consumer level but food wastage at the consumer level is not considered. (Source: UN Food and Agriculture Organization).

Three-quarters of the world’s population live in coastal areas; consequently, marine products constitute a significant proportion of protein intake in these cultures. Global seafood production is over 155 million tons per year, and the consumption of seafood is continuously increasing. Among Asia-Pacific countries, seafood consumption has increased in China, Japan, and South Korea compared to Western countries (Figure 1) [1].

Globally, over 60,000 poisoning cases per year, with a mortality rate of 1.5%, are associated with toxins produced by marine microalgae [2]. For example, in Canada, illness due to the ingestion of seafood (fish and shellfish) accounts for approximately 7% of all cases and approximately 4% of all reported cases of food poisoning [3]. The main sources of these biotoxins are phytoplankton, and the conditions for their occurrence and toxicity are not fully understood; however, several reports have shown that they are related to environmental and climatic conditions (e.g., sea surface temperature increase and ocean acidification) [4,5,6,7,8,9,10].

Seafood poisoning is caused by marine biotoxins that are naturally produced during HABs. When the environmental and climatic conditions favorable for growth coincide, phytoplankton species, mainly diatoms or dinoflagellates, grow exponentially and release harmful toxins. This mass proliferation and aggregation of some dinoflagellate species form blooms which can turn the water red or brown. Although the cause of HABs has not been clearly identified, the unexpected incidence of HABs in marine and freshwater ecosystems is increasing due to human industrial and social activities as well as climate change [11]. The HABs formed by toxin-producing phytoplankton cause significant economic losses and affect public health, commerce, fishing, tourism, and recreational activities. A paralytic shellfish poisoning (PSP) incident in New England, 2017, caused by a HAB of proliferating phytoplankton, resulted in losses of USD 120,000 to USD 20,000,000 [12]. Additionally, continued PSP poisoning of Alaskan seafood is estimated to cause economic losses of USD 6 million per year, thereby leading to adverse effects on the seafood industry, such as loss of fish and shellfish resources, quarantined coastal areas, and increased costs of monitoring [12]. Shellfish, such as mussels, oysters, and clams, are filter feeders that can accumulate biotoxins via the food chain. In most cases, toxic chemicals produced by specific photosynthetic or heterotrophic microalgae are transmitted to mollusks and are retained by filters in their digestive system, thus posing a threat to consumers [13]. Although most of these toxins are non-proteinaceous, they have varying molecular weights and exhibit unique chemical and biological properties. Consumer exposure to these toxins depends on the quantity of toxins present in the fish and shellfish consumed [14].

**Figure 2:**
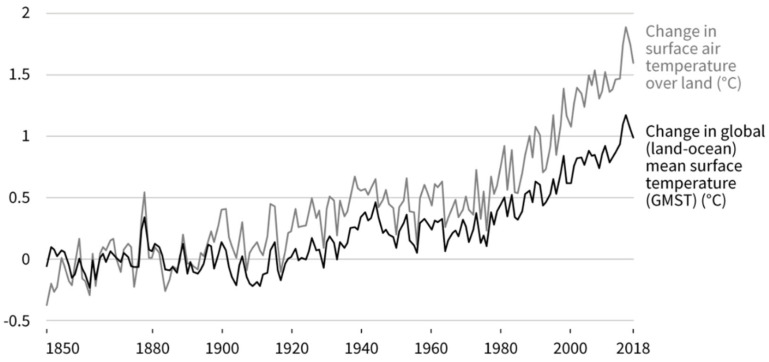
Observed temperature changes over the 1850–2018 period. Following the preindustrial period (1850–1900), the observed mean land surface air temperature has risen considerably in comparison with the global mean surface (land and ocean) temperature.

Climate change is occurring at an unprecedented rate and affects ecosystems worldwide [15]. The Intergovernmental Panel on Climate Change predicts that a sustained increase in atmospheric CO_2_ concentration will contribute to global warming by the end of the 21st century, thereby increasing the mean ocean temperature globally (Figure 2) [16,17,18]. In addition to climate change, anthropogenic activities have accelerated the rate and extent of enrichment of pollutants in many aquatic environments. These changes have a significant effect on phytoplankton, which form the base of the aquatic food chain. Mass proliferation and aggregation of microalgae is called HAB, and some species of dinoflagellates turn the sea to red or brown. In recent decades, some phytoplankton species have become increasingly complex [19,20]. The increased global incidence of HABs is often due to eutrophication in coastal areas. Changes in nutrient load, proportion, and composition have a profound effect on phytoplankton communities in rivers, estuaries, and coastal areas [19,21]. In addition, ocean temperature changes and other associated climatic changes can lead to the expansion of ecological gaps in many HAB-forming species [7,11,22].

### 1.2. Marine Biotoxins

Marine biotoxins are naturally occurring chemicals produced by certain types of toxic microalgae. Exposure to these chemicals may occur via direct contact during swimming, inhalation of droplets containing aerosolized toxins, or consumption of toxin-contaminated seafood. Illness occurs when people consume contaminated seafood, such as bivalve mollusks (e.g., scallops) or gastropods in which marine biotoxins have accumulated. The symptoms of food poisoning vary depending on the type of toxin. Marine biotoxins can be categorized as hydrophilic and hydrophobic depending on their solubility, and as paralytic shellfish poisoning (PSP), amnesic shellfish poisoning (ASP), diarrhetic shellfish poisoning (DSP), neurotoxic shellfish poisoning (NSP), and ciguatera fish poisoning (CFP) according to resultant symptoms [22,23]. Depending on their chemical structure, marine biotoxins are classified into azaspiracid, brevetoxin, cyclic imines (CIs), domoic acid, okadaic acid, pectenotoxin, saxitoxin, the yessotoxin subfamily, palytoxins (PlTXs), and ciguatoxin (Table 1) [24,25].

**Figure 3:**
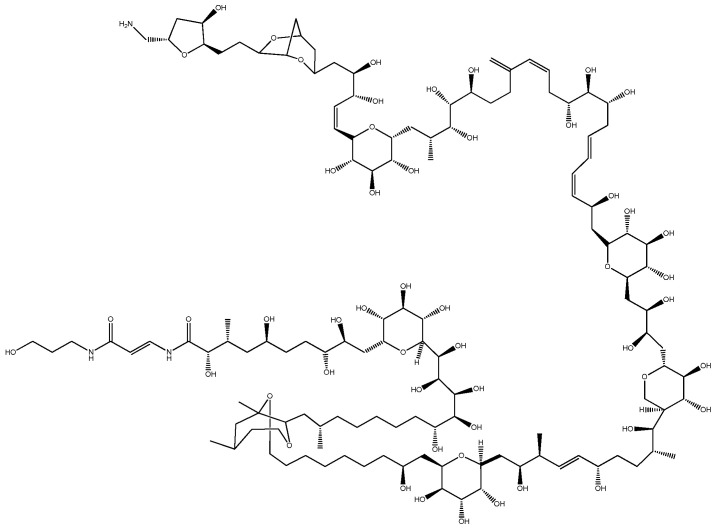
Structure of the palytoxin isolated from the soft coral *P. toxica*.

Among non-proteinaceous natural toxins, PlTX has one of the highest molecular weights (~2700 Da, Figure 3) and a very complex structure. The PlTX family comprises approximately 25 members: PlTX, 42-hydroxy PlTX (two isomers), homo-PlTX, bis-homo-PlTX, deoxy-PlTX, neo-PlTX, ovatoxins a to k, ostreocins B and D, and mascarenotoxins a and b [26,27,28], which have been found in a diverse array of marine organisms, including soft corals (e.g., *Palythoa* spp., *Zoanthus* spp., and *Parazoanthus* spp.), benthic phytoplankton, dinoflagellates (*Ostreopsis* spp.), and cyanobacteria (*Trichodesmium* spp.) [26].

Cyclic imines (Cis) are shellfish toxins related to the neurotoxin of dinoflagellates [29,30,31,32]. They are classified into four subclasses: spirolides (SPXs), gymnodimines (GYMs), pinnatoxins (PnTXs), and pteriatoxins (PtTXs). The general chemical structure of a CI toxin is characterized by a macrocycle, with a ring size between 14 and 27 carbons, associated with two subunits, the CI group (which is most often is spiroimine), and a spiroketal ring system.

SPX were first detected in mussels (*Mytilus edulis*) and scallops (*Placopecten magellanicus*) collected in Nova Scotia. SPX-B and -D were identified by nuclear magnetic resonance spectroscopy and mass spectrometry (MS) analysis, and SPX-A, -C, and 13-desmethyl SPX-C were similarly identified several years later [33].

A GYM has a six-membered CI moiety and no methyl substituents as part of the spiroimine ring system. It was first isolated from oysters and dinoflagellates in New Zealand in 1995 [34]. The first GYM subtype to be isolated has a methyl group at position C17, and is called GYM-A. Subsequently, GYM-B and GYM-C were isolated and found to exist in small amounts in *Karenia selliformis* [35].

A PnTX was first isolated from *Pinna* sp. from the South China Sea in 1990. Over the years, various forms have been isolated from *Pinna muricata* in Okinawa (PnTX A-D) [36,37] and Pacific oysters (*Crassostrea gigas*) inhabiting South Australia (PnTX E-G). Among them, PnTX-E and -G have a structure similar to that of PnTX-D, whereas PnTX-G is structurally similar to PnTX-A and -C [38].

A PtTX was first isolated from the Okinawan bivalve *Pteria penguin*. Its chemical structure is similar to that of PnTXs A–C, with the only difference being the functional group substituted at C33 [39].

Most CI toxins were discovered in shellfish prior to being found in dinoflagellates. This large and diverse taxon of eukaryotic microalgae are important primary producers of CI toxins and major causative organisms of HABs in marine ecosystems. Extensive research on dinoflagellate toxins has been conducted in recent decades, and the main poisoning syndromes caused by their toxins are PSP, NSP, ASP, DSP, and CFP. The most common species known to be involved in the production of CI toxins are *Karenia* spp., *Alexandrium* spp., and *Vulcanodinium* spp.

**Figure 4:**
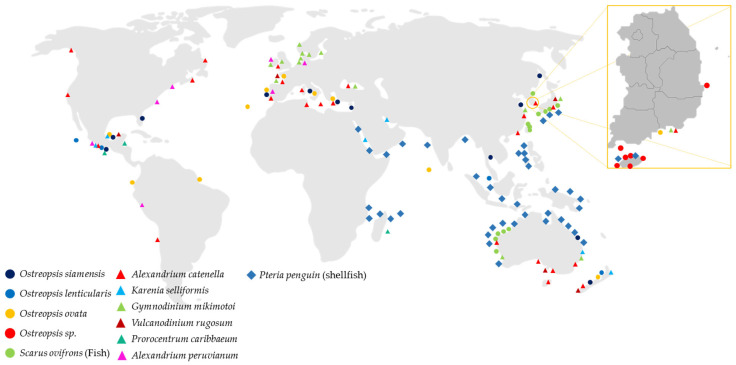
World distribution of toxic marine species. These species’ geographic ranges were retrieved from AlgaeBase.org and Aquamaps.org. These species create HABs, and their toxins accumulate in upper-food-web animals, such as fishes, shellfishes, and finally humans who consume these seafoods.

As mentioned above, although most seafood toxins originate from phytoplankton, shellfish accumulate these toxic microalgae in their bodies as they possess a filter-feeding system. CIs are found more often during HABs, and there are cases where they accumulate in shellfish; however, a direct involvement in human poisoning has not yet been proven [39,40,41]. However, in Europe, the EU Community Reference Laboratory for Marine Biotoxins of the European Food Safety Authority (EFSA) proposed a maximum allowable value of 400 μg/kg of body weight for sum of SPXs/kg shellfish meat [42]. To minimize the risk of acute poisoning from consumption of such contaminated marine animals, governments should implement appropriate monitoring programs, establish detection methods, and set regulatory limits. Phytoplankton species producing such unregulated biotoxins have been found in the Korean coastal area. Figure 4 shows that CIs or PlTX-producing phytoplankton have been found not only in Europe, but also in the East Sea of Korea, the East Sea of China, and the Philippine Sea south of Japan. The EFSA published reports in 2009 and 2010 that warn of the risks of CIs and PlTX. However, less research on the management of these toxins has been conducted in Asia-Pacific countries, including Korea. It is therefore essential to establish preemptive management of these toxins in these countries.

## 2. Phytoplankton-Derived Toxins

Of the approximately 5000 known species of marine phytoplankton, approximately 40 species (mainly dinoflagellates) produce potent toxins [6]. They have a detrimental effect on humans when we ingest tissues of toxin-accumulating crustaceans or mollusks. High mortality associated with shellfish toxins can be found in various species such as gastropods, crustaceans, and other animals in the marine food chain [2,41]. These toxins can accumulate through the food chain in higher organisms and can be a direct threat to human consumers. Once contaminated, some shellfish species cleanse biotoxins quickly, whereas others store toxins for months or years in the digestive tract and/or gonads. In many cases, the causative toxin does not originate from the crustacean such as shellfish but is rather produced by certain photosynthetic or heterotrophic microalgae and can accumulate in crustaceans at high concentrations via filter feeding [13]. For example, mussels filter 20 L of water per hour, but in the event of an HAB, millions of ingested phytoplankton cells result in accumulation of large amounts of toxins in the shellfish body [42]. Outbreaks of marine toxin poisoning are caused by the ingestion of contaminated shellfish and may induce a wide range of symptoms associated with certain toxic compounds [43,44]. Some dinoflagellate species such as *Noctiluca scintillans* and *Skeletonema costatum* enriched by an HAB are associated with water discoloration and death of marine organisms but are not related to shellfish toxins. However, some of them are known to be the major causative organisms of shellfish poisoning such as *Alexandrium*, *Gymnodinium*, *Dinophysis,* and *Pseudo-nitzschia* [45,46].

According to some studies, the HAB phenomenon is worsening due to environmental pollution, aquaculture expansion, overnutrition, and species migration caused by cyst formation and the ballast water of large vessels [47,48,49]. Furthermore, *Alexandrium ostenfeldii*, found in subtropical areas, and especially *Ostreopsis* spp., have been found on Jeju Island and the south coast of South Korea [50,51]. For this reason, it is necessary to pay attention to marine biotoxins that are not regulated and to set up countermeasures against them.

### 2.1. Ostreopsis—PlTX and Its Analogs

*Ostreopsis* cf. *ovata* is widely distributed in tropical and temperate climate regions and has been reported to persist year-round in Pacific tropical waters. Moreover, seasonally varying patterns of HABs are observed in the Mediterranean, which often coincide with the high temperature of the sea [52,53,54]. In several laboratory studies, the optimum temperature for the growth of *O.* cf. *ovata* from the Mediterranean Sea has been found to be 22 °C [55,56], and for *O.* cf. *ovata* from the Adriatic Sea, it was found to be 20 °C [57]. Moreover, according to a study by Yamaguchi et al., high salinity and temperature of seawater affect the growth of *O.* cf. *ovata* [58]. Ocean temperature in the Asia-Pacific region is increasing, and this change is beneficial for the growth of *Ostreopsis* spp. Furthermore, the probability of PlTX detection is also increasing [59].

The *O.* cf. *ovata* strain found in the Mediterranean Sea is known to produce PlTX and its analogs ovatoxin-a, -b, -c, -d, -e, -g, and -h [26,60,61,62,63,64]. For *O.* cf. *ovata*, it has been confirmed that PlTX accumulates in the cells, but there have been no cases of poisoning by ingestion of contaminated marine products from the same area [65,66,67,68]. However, there have not only been several cases of inhalation of aerosols or direct exposure of the skin, but also massive deaths of invertebrates caused by toxins. The toxin content in *O.* cf. *ovata* is inversely proportional to its growth rate, indicating that a larger amount of the toxin is produced under unfavorable conditions [58,60,69].

Other pathways through which PlTX accumulates also exist (Table 2). Poisoning cases related to PlTX-containing seafood have been reported in tropical and subtropical regions, and there are reports on the correlations of *Ostreopsis* and PlTX exposure pathways based on soft coral trade for aquarium decoration purposes [70]. Another route is fish ingesting *Ostreopsis* spp. or accumulation of *Ostreopsis* in fish while feeding on algae to which *Ostreopsis* spp. are attached. According to the results of the study by Taniyama et al., convulsions and drowsiness linger for a long time in rats exposed to a *Ostreopsis* sp., consistently with the initial symptoms of PlTX poisoning [71]. In addition, delayed hemolysis after a reaction of an anti-PlTX antibody or ouabain—in an experiment with an *Ostreopsis* sp. fed to the herbivorous parrotfish *Scarus ovifrons*—was found to be consistent with the results on PlTX. In that study, the herbivorous parrotfish *S. ovifrons* and a serranid *Epinephelus* sp. ingested an *Ostreopsis* sp., a dinoflagellate that produces PlTX, which accumulates in the body and causes poisoning symptoms [71,72].

The most characteristic symptom of patients with PlTX poisoning is severe myalgia due to rhabdomyolysis, which is usually accompanied by abnormally elevated levels of myoglobin, urea, and serum creatine phosphokinase (CPK) [72]. According to Suzuki et al., 27 cases of poisoning by ingestion of *S. ovifrons* occurred from May 1953 to October 2011, and 5 out of 94 patients died [75]. In 1986, a poisoning incident involving *Ypsiscarus ovifrons,* one of the scaly sea breams, occurred in Japan, and as a result of analyzing the fish, it was reported that it was poisoned by PlTX [73].

The chemical structure of PlTX was revealed by Moore and Bartolini in 1981 [76]. PlTX is one of the largest microalgal toxins (molecular weight 2680 Da) among nonpolymeric natural compounds and has a very complex structure. Its chemical formula is C_129_H_223_N_3_O_54_, and the compound is water-soluble and contains 64 chiral centers.

PlTX is known to be the most potent toxin among nonproteinaceous marine toxins. The first report in this field was about *Palythoa toxica*, a soft coral inhabiting the Hawaiian coast, and PlTX has since been found in several *Palythoa* species [76]. PlTX and its analogs accumulate in primary consumers such as fish and crustaceans (e.g., shellfish) via the food chain. Humans at the top of the food chain show symptoms of poisoning after ingestion, skin exposure, or inhalation. The known PlTX toxicity mechanism keeps both gates of the Na^+^/K^+^ ATPase pump open at the same time while inhibiting the activity of nicotinic acetylcholine receptors; consequently, Na^+^ flows in and K^+^ flows out of the cell persistently, which induces depolarization, eventually having toxic effects [77,78]. When PlTX is ingested, the major symptoms are gastrointestinal problems, muscle pain, cardiac dysfunction, respiratory problems, and cyanosis. Although there are no substantive reports on acute poisoning, the CONTAM Panel was only able to derive an oral acute reference dose (ARfD) of 0.2 μg/kg b.w. for the sum of PlTX and its analogue ostreocin-D [79]. The half-maximal lethal dose (LD_50_) values for PlTX, as determined in animal experiments, are shown in Table 3.

### 2.2. CIs: SPXs, PnTXs, GYMs and PtTXs

**Figure 5:**
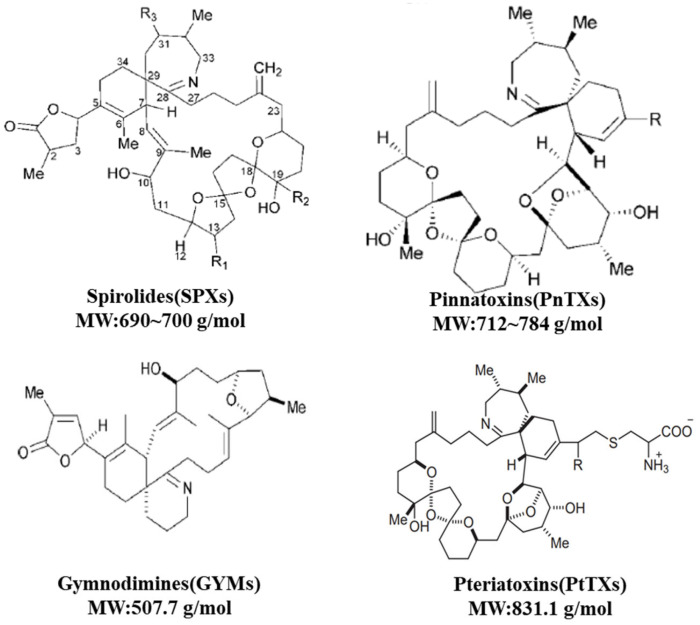
Structures of CIs isolated from various phytoplankton species.

CIs are “fast-acting” toxins associated with red tides and shellfish toxicity [88]. Their chemical structure commonly consists of 14–27 carbon atoms and has three characteristics: a giant ring, a ring-shaped imine group, and a spiroketal ring system. CIs contain 5–7-membered rings (SPXs, PnTXs, and PtTXs) and, in most cases, one or two methyl groups. The spiroketal ether ring system can be a simple tetrahydrofuran (e.g., portimine or GYMs), a tetrahydrofuran group (e.g., spiro-prorocentrimine or prorocentrides), or more complex 6,5-SPXs (H and I), 6,6,5-SPX G, 6,5,5-SPXs A–F, or 6,5,6-spiroketals (PnTXs or PtTXs). These toxins were first found in plankton and shellfish tissue extracts. CI toxins can be classified into GYMs, PnTXs, PtTXs, and SPXs (Figure 5) [92,93]. Rundberget et al., and Tillmann et al., have reported that the structures of 36 CI toxins have been identified by means of mass spectrometric data, and it can be predicted that there may be more toxins, with greater structural diversity [94,95]. The dinoflagellate *K. selliformis* produces only a GYM, whereas some strains of *A. ostenfeldii* produce both a GYM and SPX. [96,97]. Van Wagoner et al., suggested that such structural similarities are due to similar groups of genes involved in their biosynthesis; SPXs and GYMs are produced from linear initial polyketide chains formed by the attachment of small units such as acetate and glycine [98].

#### 2.2.1. *A. ostenfeldii*—SPX and GYM

A causative phytoplankton species associated with SPX and GYM (described in Section 2.2.4) accumulation was found in cultured shellfish in Nova Scotia and identified as a mixotrophic *Dinophyceae* member: *A. ostenfeldii* [99]. This species is widely distributed in coastal waters around the world, including the Washington coast, northeastern coast of North America, North Atlantic Ocean, Arctic Ocean, and Mediterranean and Baltic Seas [42,100,101]. Van de Waal et al., investigated paralytic shellfish toxin-associated poisoning caused by an HAB in the Netherlands and reported that this phenomenon was not related to human poisoning by SPXs [102]. The regulation of biosynthesis of these toxins is controlled by complicated mechanisms because certain complex species, e.g., *A. ostenfeldii* and *Alexandrium peruvianum*, produce PSP and/or CI toxins. Therefore, it is difficult to predict and evaluate the profile and levels of toxins because they differ among species strains with different geographical origins [103].

An SPX is a polyether CI toxin with a spiro group attached to the tricyclic ether. These toxins were first isolated from mussels, and toxicity data were collected using a mouse bioassay (MBA) during chemical analysis of polar bioactive molecules in phytoplankton and crustaceans of Nova Scotia (Canada) [104]. The CI residues of these toxins are known as pharmacophores with biological activity [80]. There are SPXs a, b, c, d, and desmethyl derivatives, and they represent “fast-acting” toxins affecting muscarinic and nicotinic acetylcholine receptors [81] as well as exerting toxicity through Na^+^/K^+^ ATPase channels and an irreversible action on weak L-type transmembrane Ca^2+^ channels [82]. As a result of intraperitoneal (i.p.) administration of an SPX in MBAs, such symptoms as abdominal cramps, hyperextension, and tail bending can occur [83]. The LD_50_ values of SPX-E and -F are higher than those of SPX-B and -D, respectively, and the LD_50_ value was confirmed to be 40 μg/kg b.w. when a mixture of SPXs was administered [84].

#### 2.2.2. *Vulcanodinium rugosum*—PnTXs

*V. rugosum*, a dinoflagellate isolated from waters near New Zealand, is known to produce a PnTX [105]. This species was officially isolated for the first time in the Mediterranean lagoon and since then has been found in Australia, New Zealand, Japan, Hawaii, and Europe [90,91].

A PnTX was first isolated from an extract of the crustacean *Pinna attenuata* Reeve collected in Guangdong, China, in 1858 [106]. PnTXs are a subfamily of CI toxins, and PnTX-A was the first to be analyzed [107]. PnTXs B–D have been isolated from Japanese *P. muricata* [37,85], and PnTXs E–G have been isolated from shellfish in Northern New Zealand and South Australia [38,108]. PnTX-G was also found in Norwegian [85] and Canadian mussels, suggesting that this toxin is distributed globally [94,101]. Although MBAs have revealed that PnTXs are toxic (to rodents), there are no human studies to confirm these findings [38,108]. Results of an acute toxicity study on a PnTX administered by i.p. injection are shown in Table 3. LD_50_ values ranged from 16 to 50 μg/kg b.w., with PnTX-E and -F showing the strongest toxicity [38,109]. Mice given a lethal dose of a PnTX showed such symptoms as sudden inactivity and dyspnea after 10 min of overactivity. Mice given sublethal doses of a PnTX became lethargic approximately 9 to 13 min after administration but recovered within 2 h when respiration was maintained normally [38].

#### 2.2.3. *P. penguin*—PtTX

A PtTX was found in extracts of shellfishes *P. penguin* and *P. muricata* [85]. *P. penguin,* also known as the penguin wing oyster or wing shellfish, inhabits the western and central regions of the Indo-Pacific region and is distributed along the East African coast, the Red Sea, India, southern China, southern Japan, the Philippines, Indonesia, and northern Australia. *P. muricata* is a bivalve mollusk belonging to the family Pinnidae. It is known as a major source of sea silk, is distributed worldwide, and is believed to have existed since the Jurassic period [87]. According to a study by Takada et al., pteriatoxins (A-C) were assumed to have the same absolute stereochemistry of PnTXs [86]. Selwood and colleagues extracted PnTXs from razor fish and discovered two very different kinds of PnTX, called PnTX-F and PnTX-G [38]. From the results, they suggested that PtTX is a metabolite of PnTX-G and their metabolic processes appear to occur in Pacific Oysters, such as *P. penguin* and *P. muricata* [38]. PtTX is also a fast acting toxin, and LD_99_ of PtTX-A was found to be 100 μg/kg b.w., and that of a PtTX-B and -C mixture is 8 μg/kg b.w. [86].

#### 2.2.4. *Karenia* spp.—GYM

*Karenia* species, which are harmful microalgae affiliated with marine dinoflagellates, are found worldwide. These species are known to produce toxins such as brevetoxin, GYMs, and ichthyotoxin, which may cause a disease or even death in humans and marine animals [89]. *Gymnodinium mikimotoi*, found in Japan, is known to be associated with the death of fish and oysters and was renamed *Karenia mikimotoi* [90]. *K. brevis* is one of the most studied harmful algae because of extensive investigation of its toxicity, physiology, and HABs (e.g., HABs in Florida), which cause widespread marine animal deaths and have detrimental effects on human health [98,110,111]. Over the past 50 years, the frequent HABs in the Gulf of Mexico under the influence of *K. mikimotoi* and *K. brevis* have resulted in mass deaths of marine animals, and neurotoxic shellfish toxins and dyspnea are considered a major problem. It has been discovered that some *Karenia* species produce various toxic sterols such as GYMs and gymnocin, and polyunsaturated fatty acids (e.g., octadecapentaenoic acid), and it is possible that new *Karenia* species and toxins will be discovered in the future [89].

A GYM was first isolated from the Foveaux Strait oyster [34] and is a toxin that contains a spiro center and imine functional groups; GYMs are structurally similar to PnTXs, SPXs, and PtTXs [93,109,112]. Later, a GYM was discovered in shellfish found along the coasts of New Zealand, Tunisia, and Canada.

GYMs have shown a lethal dose of 80–96 μg/kg b.w. after i.p. injection into female rats. Symptoms of acute toxicity of GYMs A and B were hyperactive behavior, jumps, slow movements, and numbness. After that, the rats became immobile and unresponsive to a stimulus. At nonlethal doses, dyspnea was noted, but recovery to normal status was observed within 30 min with no squeals [88].

## 3. Analytical Protocols for Novel Marine Biotoxins

The MBA is commonly used as a shellfish toxicity monitoring tool in most countries; it was standardized by the Association of Official Analytical Chemists (AOAC; official method: AOAC 959.08) for quick, reliable, and accurate measurements [113]. The advantage of this analysis is that it can be used to detect multiple toxins in a wide range of organisms such as mollusks and crustaceans, and it is a formal analytical method used to assay regulated-toxin levels in seafood in most countries. However, the disadvantages of this assay include mouse supply problems, unsatisfactory limits of quantitation and detection, a nonlinear dependence of death time on toxin levels (positive) and killing of many animals. Cell-based assays are being developed to replace the MBA, but problems such as interference by other shellfish toxins still exist [79,113].

Lately, liquid chromatography coupled with tandem mass spectrometry (LC-MS/MS) has been used as a toxin assay (Table 4). In this assay, peak selectivity and accuracy are higher than those of other analytical methods, and therefore multiple components can be analyzed simultaneously, helping to detect multiple toxins in one sample. In addition, trace analysis is possible, and the assay time is shorter than that of the MBA; accordingly, LC-MS/MS has attracted attention as a toxin detection method. However, it is difficult to obtain a standard substance, and if the amount of toxins in a sample is low, quantitative analysis is performed using external standard substances.

Owing to the development of this instrumental analytical method, LC-MS/MS or LC coupled with high-resolution MS (HRMS) has been mainly used in recent studies on PlTX and CI subfamilies.

In case of PlTX, which is a water-soluble toxin, 50% methanol is used as a solvent for extraction. In other studies, a small amount of acetic acid (0.1%) has been added to methanol [116]. In a study by Beress et al., PlTX was extracted using 50% ethanol, the cleanup step was conducted using charcoal, and PlTX was analyzed by column chromatography. However, recently, the solid-phase extraction (SPE) method was mainly used at the cleanup step. Strata-X, Oasis HLB, and the C-18 column are mainly used for LC-MS/MS analysis [114,117]. In most cases, water and acetonitrile are mainly used as mobile phases, and the analysis is performed by adding a small amount of acetic acid or formic acid (0.1%).

For the analysis of CIs, which are hydrophobic toxins, 100% methanol or methanol with 0.05% formic acid have been used as extraction solvents. For sample cleanup, the SPE method has been used, e.g., with the Strata-X, Oasis HLB, or C18 cartridge. LC-MS/MS, LC-HRMS, and ultra-high-performance LC (UPLC)-MS typically are based on C18 columns, mainly for instrumental analysis. Water containing formic acid (50–53 mM) or ammonium formate (2–3.66 mM) and acetonitrile (properties similar to those of water) have been used as mobile solvents.

For the instrumental analysis as described above, the sensitivity of the assay may vary greatly depending on the analytical conditions, such as the composition of the sample added to the mobile phase, the program of the solvent gradient, and the type of column. Hence, it is important to establish standardized conditions for toxin analysis.

PlTX and CI toxins have different solubility properties, but several studies have confirmed that 50–100% methanol can be used for the initial extraction (Table 4). It is possible to analyze two toxins at the same time if analytical conditions such as cleanup and/or column are standardized. Therefore, it is necessary to develop and optimize an efficient, fast, and accurate multiplex analytical method for hydrophilic/lipophilic toxins.

## 4. Conclusions

Global warming has led to an increase in average temperatures worldwide, and ocean temperatures are also increasing. These changes increase the incidence of HABs caused by phytoplankton, resulting in a lack of oxygen and destruction of marine ecosystems, such as fish and shellfish habitats. The number of phytoplankton species that produce toxins has also increased. In addition to the increased temperature of the ocean, *Ostreopsis* spp., *Vulcanodinium* spp., and *Alexandrium* spp. inhabiting warm climatic zones and producing toxic compounds have been discovered on the coast of Korea as they migrate from tropical to temperate regions. This process causes the accumulation of toxins in shellfish (e.g., scallops) in the new region and exposes people to uncomfortable experiences after ingestion of these seafoods. This leads to such symptoms as diarrhea, muscle cramps, and dyspnea in humans, who are quaternary consumers in the food chain. The toxins found in phytoplankton are CI toxin subfamilies such as SPXs, PnTXs, PtTXs, and GYMs. These CI toxins have been reported to be more toxic than regulated toxins such as shellfish toxins (PSP, NSP, ASP, and DSP). There have been no cases of human poisoning in Korea. As the habitat of toxin-containing phytoplankton is expanding, the causative phytoplankton species are becoming more common in Korean coastal areas. The geographic ranges of these causative phytoplankton species are displayed in Figure 5. Most of the species are distributed in the seas of the world and occur in countries of the Asia-Pacific region, including Korea. Therefore, preemptive management of these toxins must be established by experts on toxins or on analytical methods.

## Figures and Tables

**Table 1:** Classification of marine biotoxins and the key adverse effects in humans.

Type of Poisoning	Marine Biotoxins	Source	Symptomatology
PSP	Saxitoxin	*Alexandrium* spp. *Gymnodinium catenatum* *Pyrodinium bahamense*	Gastrointestinal symptoms Paralytic phenomena Recovery or death
ASP	Domoic acid	*Pseudo-nitzschia* spp. *Nitzschia* spp.	Gastrointestinal and neurological symptoms Cardiac or respiratory problems Recovery or death
DSP	Okadaic acid	*Prorocentrum lima* *Dinophysis* spp.	Gastrointestinal symptoms Recovery within 3 days
	Dinophysistoxins	*Dinophysis acuminata* *D. caudata*	nausea, vomiting, severe diarrhea
NSP	Brevetoxin	*Karenia brevis*	Gastrointestinal and neurological symptoms, respiratory problems, Recovery or death
AZA	Azaspiracid	*Amphidoma languida* *Azadinium spinosum*	Gastrointestinal symptoms
YTX	Yessotoxins	*Proroceratium reticulatum* *Lingulodinium polyedrum* *Gonyaulax spinifera*	Lack of observations in humans
CFP	Ciguatoxin	*Gambierdiscus* spp.	Gastrointestinal symptoms, cardiovascular or neurological problems
CIs	Spirolides	*Alexandrium* spp. *Karenia* spp. *Vulcanodinium* spp. *Prorocentrum* spp.	Lack of observations in humans
	Pinnatoxins	*Vulcanodinium rugosum* *Pteria penguin* *Pinna muricata*	
	Pteriatoxins		
	Gymnodimines	*Karenia selliformis* *(Gymnodinium selliformis)* *Alexandrium peruvianum* *Alexandrium ostenfeldii*	
PlTXs	Palytoxin Palytoxin-b 42-hydroxy-palytoxin Homo-palytoxin Bis-homo-palytoxin Neo-palytoxin Deoxy-palytoxin Mascarenotoxin-a, Mascarenotoxin-b, Mascarenotoxin-c Ovatoxin-a, b, c, d, e, f, g, h, i, j, k Ostreocin-a, b, d, e	*Palythoa caribaeorum* *Palythoa toxica* *Ostreopsis lenticularis* *Ostreopsis siamensis* *Ostreopsis ovata* *Ostreopsis mascarenensis*	Gastrointestinal symptoms Muscle and cutaneous problems

**Table 2:** Cases of human poisoning through fishes containing PlTXs.

Species	Localization	Toxins	References
*Scarus ovifrons*	Japan	Palytoxin-like toxins	[71]
*Calotomus japonicus*	Japan	Palytoxin-like toxins	[69]
*Epinephelus* sp.	Japan	Palytoxin-like toxins	[72]
*Ypsiscarus ovifrons*	Japan	Palytoxin	[73]
*Herklotsichthys quadrimaculatus*	Madagascar	Palytoxin and its analogue	[74]

**Table 3:** Lethal doses of marine biotoxins.

Toxins	Route	Lethal Dose	Refs.
Palytoxins	i.p.	i.p. injection into mice, LD_50_: − palytoxin: 50 ng/kg − ostreocin-D: 750 ng/kg − mascarenotoxin-A: 900 μg/kg	[77,78,79]
Spirolides	i.p.	i.p. injection into mice, LD_50_: − spirolide A: 37 μg/kg − spirolide B: 99 μg/kg − spirolide C: 8 μg/kg − 13-desmethyl spirolide C: 7–28 μg/kg − 13,19-didesmethyl spirolide C: 32 μg/kg − 27-Hydroxy-13-desmethyl spirolide C: >27 μg/kg − 27-oxo-13,19-didesmethyl spirolide C: >35 μg/kg − spirolide E: >1000 μg/kg − spirolide F: >1000 μg/kg − 20-methyl spirolide G: 63 μg/kg	[80,81,82,83,84]
	Oral	Oral administration to mice, LD_50_ (μg/kg): − spirolide mixture (gavage): 1000 − spirolide C (gavage): 176 − spirolide C (fed on cream cheese): 780	
Pinnatoxins	i.p.	i.p. injection into mice, LD_99_ (μg/kg): − pinnatoxin A: 135–180 − pinnatoxin B and C: 22 − pinnatoxin D: 400 − pinnatoxin H: 67 − i.p. injection into mice, LD_50_ (μg/kg): − pinnatoxin E: 33.5–75.3 − pinnatoxin F: 9.5–15.8 − pinnatoxin G: 35.0–68.1	[39,85]
	Oral	Oral administration to mice, LD_50_ (μg/kg): − pinnatoxin E (gavage): 2380–3000 − pinnatoxin F (gavage): 19.1–35.1 − pinnatoxin F (16 h fased): 37.9–71.5 − pinnatoxin G (gavage): 105–199 − pinnatoxin G (cream cheese mixture): 380–470	
Pteriatoxins	i.p.	i.p. injection into mice (LD_99_): − pteriatoxin A: 100 μg/kg − pteriatoxin B/C: 8 μg/kg	[86,87]
Gymnodimines	i.p.	i.p. injection into mice, LD_50_ (μg/kg) − gymnodimine A: 79–118 − gymnodimine B: 800	[88,89,90,91]
	Oral	Oral administration to mice LD_50_ (μg/kg): − gymnodimine A (gavage): 600–945 − gymnodimine A (voluntary feeding on mouse food): >7500	

**Table 4:** Methods for extraction, cleanup, and LC-based analysis of PlTXs and/or CIs from marine organisms.

Sources	Target Toxins	Extraction	Clean up or Purification Process	Instruments	Column	Mobile Phase	Refs.
*Chondria armata*	PlTX	4 L of water below 10 °C	DEAE-cellulose column (10 × 5 cm, OH^-^ form) → LiChroprep RP-18 column (5 × 5 cm) → TSK G3000S column (1 × 7.5 cm).	Orbitrap Elite FT mass spectrometer	Reverse-phased column, Develosil C30-UG-3 2.0 i.d. ×100 mm	A: 0.1% acetic acid B: 0.1% acetic acid containing acetonitrile Gradient: 10–100% B for 23 min, minutes 25–26 100–10% B, minutes 26–30 10% B	[114]
Mussel	PlTX	Methanol (MeOH)–H_2_O 8:2 (*v/v*)	SPE (Strata-X, Strata-XL, OASIS HLB LP 6 cc, PolyLC INC) 1. Load: MeOH–H_2_O 2:8, 1:9, 5:95 2. Wash: MeOH–H_2_O 1:1, 4:6, 3:7, 1:9, H_2_O 100% 3. Elute: MeOH 100%, MeOH–H_2_O 9:1, 8:2, 8:2 with 0.2% acetic acid, MeOH with 1% acetic acid, MeOH– H_2_O 8:2 with 0.1% trifluoroacetic acid, isoPrOH–H_2_O 8:2, isoPrOH–H_2_O–acetic acid 40:59:1, 70:29:1.	LC-HRMS	1. Gemini C18, 3 μm, 2 × 150 mm 2. Kinetex C18, 2.6 μm, 2.10 × 100 mm 3. Poroshell 120 EC-C18, 2.7 μm, 2.1 × 100 mm	A: H_2_O, 30 mM acetic acid B: 95% MeCN–H_2_O, 30 mM acetic acid	[115]
*Palythoa caribaeorum*	PlTX	50% EtOH	Charcoal	Gel filtration	1. Sephadex G-S0 Column, 7 × 150 cm	0.1 M acetic acid	[115]
				Gel filtration	2. QAE-Sephadex A-25 Column, 3 × 30 cm	0.01 M Tris-HCl, pH 8	
				Gel filtration	3. SP-Sephadex column C-25, 1 × 60 cm	0.01 M Na-acetate solution, pH 4.5	
				Ion exchange chromatography	4. CM-Cellulose column, 1.5 × 30 cm	0.005 M NH_4_-acetate buffer	
				Gel filtration	5. Biogel P-6 column (200–400 mesh)	0.1 M acetic acid	
*Ostreopsis siamensis*	Ostreocin-D	MeOH followed by MeOH-H_2_O-AcOH (50:50:0.1)	Partitioning with CHCl_3_ and using aqueous part for further purification	Column chromatography	1. Develosil Lop ODS column	MeOH-H_2_O-AcOH (90:10:0.2)	[116]
					2. Develosil TMS-5 column	MeCN-H_2_O-AcOH from 22:78:0.1 (20 min) to 25:75:0.1 (30 min)	
*Palythoa, Protopalythoa, or Zoanthus* spp.	PlTXs (hydrophilic toxins)	Water → homogenization → ultrasonication (320 W)	Add chloroform:MeOH (2:1) → separate aqueous layer (palytoxin) → SPE (1 mL C-18-T (Phenomenex, Torrance, CA, USA)	LC-MS/MS	1. 50 × 2.1 mm Kinetex column (2.6 µm particle with C-18 from Phenomenex) 2. 50 × 2.1 mm HILIC column (3.5 µm particle from Millipore)	A: water (0.1% formic acid) B: acetonitrile (0.1% formic acid) Gradient: 20% B to 80% B for 25 min	[110]
*Ostreopsis* cf. *ovata*	Ovatoxins a to e and isobaric PlTX	MeOH:water (1:1) → sonication for 10 min	-	LC-HRMS	Poroshell 120 EC-C18 (2.7 μm, 2.1 × 100 mm) column	A: water (30 mM acetic acid) B: 95% acetonitrile (30 mM acetic acid) Gradient: 28–29% B for 5 min 29–30% for 10 min, 30–100% B for 1 min, held for 5 min	[111]
*O.* cf. *ovata*	PlTX, ovatoxins a–d, mascarenotoxins a and c	MeOH:water (1:1)	0.22 μm pore size membrane filter	LC/time-of-flight (TOF)-MS	Phenomenex Luna HILIC 3μ 150 × 2.00 mm	A: water (0.1% formic acid) B: 95% acetonitrile (0.1% formic acid) Gradient: minutes 0–2 10% A minutes 2–5 50% A minutes 5–10 50% A then linear decrease from 50% A to 10% A (initial condition)	[117]
*O.* cf. *ovata*	PlTX	50% MeOH with sonication for 4 min	-	LC-HRMS	Accucore C18 column (2.6 μm, 100 × 2.1 mm; Thermo Fisher)	A: water (0.1% formic acid) B: acetonitrile (0.1% formic acid) Gradient: 26–29% B for 15 min, 29–99% B for 4 min, 99–29% B for 0.5 min, and hold for 10.5 min	[118]
*O.* cf. *ovata*	Isobaric PlTX, ovatoxins a–e, g	MeOH:water (80:20)	0.22 μm syringe filter	hybrid linear ion trap LTQ Orbitrap XL™ Fourier transform mass spectrometer (FTMS) equipped with ESI ION MAX™ source (Thermo Fisher, San José, CA, USA) coupled to Agilent 1100 LC binary system	Poroshell 120 EC-C18, 2.7 μm, 100 × 2.10 mm column	A: water (30 mM acetic acid) B: 95% acetonitrile (30 mM acetic acid) Gradient: minutes 0–10 28–29% B minutes 10–20 30% B minutes 20–21 100% B and hold for 10 min	[64]
Greenshell mussel™ (*Perna canaliculus*), Pacific oyster (*Crassostrea gigas*), Parrot fish (*Scarus ovifrons*)	PlTX	MeOH:water (1:1)	60 mg Strata-X SPE cartridge (Phenomenex, Torrance, CA, USA) Pre-conditioned: 3 mL of MeOH → 3 mL of water Wash: 2 mL of MeOH:water (2:3) → 2 mL of water	LC-MS/MS	Acquity C18 HSS 1.7 μm column 50 × 1 mm	A: water (0.1% formic acid) B: acetonitrile (0.1% formic acid) Gradient: 0% B for 0.5 min 60% B for 2.5 min 100% B for 2.5–3 min (flushing)	[119]
Mediterranean mussels, European oysters, queen scallops, and ascidians	GYM, PnTX-G, SPX-C	MeOH	-	Electrospray ionization (ESI)-MS/MS	Zorbax SB-C8RRHD 2.1 × 50 mm, 1.8 μm	A: water (3.66 mM ammonium formate + 53 mM formic acid) B: acetonitrile (3.66 mM ammonium formate + 53 mM formic acid) Gradient: 30–90% B for 0.5–8 min	[120]
Mussels (*Mytilus galloprovincialis*)	GYM-A, PnTX-G	MeOH	SPE, Strata-X cartridge, 30 mg/mL	LC-MS/MS	Agilent Zorbax SB-C8 Rapid Resolution HD (2.1 × 50 mm, 1.8 µm)	A: water (2 mM ammonium formate + 50 mM formic acid) B: 95% acetonitrile (2 mM ammonium formate + 50 mM formic acid) Gradient: minutes 0–5 20–50% B minutes 5–6 50–20% B	[121]
*Patella rustica complex, Phorcus turbinatus, Spondylus spinosus*	GYM-B, SPX	MeOH	-	LC-MS/MS (Agilent Technologies 6410)	5 μm Poroshell C18, 50 × 2.1 mm Agilent column	A: water (2 mM ammonium formate) B: 95% acetonitrile (50 mM formic acid)	[122]
Shellfish	GYM-A, SPX	MeOH		LC-MS/MS (Thermo Ultimate 3000 HPLC system coupled to AB-Sciex Qtrap 4500 mass spectrometer)	Luna C18 column (50 mm × 2.1 mm Phenomenex)	A: water (2 mM ammonium formate + 50 mM formic acid) B: 95% acetonitrile (2 mM ammonium formate + 50 mM formic acid) Gradient: 5–50% B for 1 min, 50–100% B for 4 min, 100% B for 2 min.	[123]
*Mytilus edulis*	GYM-A, SPX	MeOH		ESI-MS/MS	Thermo Finnegan BDS Hypersil C8 (50 mm × 2.1 mm, 3 μm) column	A: water (2 mM ammonium formate + 50 mM formic acid) B: 95% acetonitrile (2 mM ammonium formate + 50 mM formic acid) Gradient: 30% B to 90% B for 8 min, 90% B for 2.5 min, return for 0.5 min to 30% B	[24]
					Waters X-Bridge C18 (150 mm × 3 mm, 5 μm) column	A: water (6.7 mM ammonium hydroxide) B: 95% acetonitrile (6.7 mM ammonium hydroxide) Gradient: 10% B for 1 min and increase linearly to 90% B in 9 min, keep at 90% B for 3 min and return to 10% B in 2 min	
*Alexandrium ostenfeldii*	13-desmethyl SPX-C	0.05% formic acid in MeOH		LC-MS	50 × 2.1 mm i.d., 2.5 μm Luna C18 column (Phenomenex)	A: water (2 mM ammonium formate + 50 mM formic acid) B: 95% acetonitrile (2 mM ammonium formate + 50 mM formic acid) Gradient: 10–100% B for 6 min, 100% B for 2 min, return to 10% B for 0.5 min	[124]
				LC-HRMS	2.7 μm Agilent Poroshell SB-C18 column	A: water (2 mM ammonium formate + 50 mM formic acid) B: 95% acetonitrile (2 mM ammonium formate + 50 mM formic acid) Gradient: 5–100% B for 20 min, 100% B for 5 min, return to 5% B for 1 min	
*A. ostenfeldii*	13-desmethyl SPX-C	MeOH	SPE cartridge (Waters Oasis HLB)	LC-HRMS	Poroshell 120 SB C18 column (2.1 × 150 mm, 2.7 μm)	A: water (2 mM ammonium formate + 50 mM formic acid) B: 95% acetonitrile (2 mM ammonium formate + 50 mM formic acid) Gradient: 5–100% B for 25 min	[125]
Shellfishes	CIs	MeOH		LC-MS/MS	Poroshell 120 EC-C18 column (100 × 2.1 mm, 2.7 µm)	A: 2 mM ammonium acetate and 18 mM glacial acetic acid in 5.2% methanol B: 1 mM ammonium acetate in 100% methanol Gradient: 5% B until minute 1, 63% B until minute 2, 86% B until minute 4, 100% B until minute 11	[126]
*Perna canaliculus* (green-lipped mussel) *Mytilus chilensis* (shellfish)	13-desmethyl SPX-C, PnTX-G	MeOH		UPLC-MS	Acquity UPLC BEH C18 (2.1 × 100 mm, 1.7 µm)	A: water (2 mM ammonium formate + 50 mM formic acid) B: 95% acetonitrile (2 mM ammonium formate + 50 mM formic acid) Gradient: 30–70% B until minute 3, 70% B until minute 4.5, 30% B until minute 4.6	[127]
Green mussels (*Perna viridis*), backwater oysters (*Crassostrea madrasensis*)	13-desMeC SPX, 20-Me SPX-G, GYM	MeOH	SPE	LC-MS/MS	1.7 μm, 2.1 × 50 mm Acquity BEH Amide UPLC column	-	[128]
*Mytilus galloprovincialis* and *Ruditapes decussatus*	13-desMeC SPX, GYM-G, -H, -I, -J	MeOH	-	LC-HRMS	HyperClone BDS C8 column 50 × 2.0 mm, 13 Å, 3 μm	A: water (2 mM ammonium formate + 50 mM formic acid) B: 95% acetonitrile (2 mM ammonium formate + 50 mM formic acid) Gradient: 10–100% B for 10 min, 100% B for 15 min	[129]
mussels *(Mytilus galloprovincialis),* clams *(Ruditapes philippinarum) and* oysters *(Ostrea edulis)*	13desmSPXC, GYMA, 13,19didesmSPXC, 20MethylSPXG and PnTXG And 9 other lipophilic toxins	MeOH	0.22 μm syringe filter	LC-MS/MS	Phenomenex Kinetex EVO C18 “core–shell” column 50 mm × 2.1 mm, 2.6 µm	A: water (6.7 mM NH_4_OH (pH 11)) B: 90% acetonitrile (MeCN) (6.7 mM NH_4_OH (pH 11)) Gradient: 22% B for 0.1 min, 22–95% B for 1.8 min, maintaining until 2.90 min	[130]

## Data Availability

Not applicable.

## Abbreviations

ASP: amnesic shellfish poisoning; CFP: ciguatera fish poisoning; CI: cyclic imine; DSP: diarrhetic shellfish poisoning; GYM: gymnodimine; HAB: harmful algal bloom; NSP: neurotoxic shellfish poisoning; PlTX: palytoxin; PnTX: pinnatoxins; PSP: paralytic shellfish poisoning; PtTX: pteriatoxins; SPX: spirolide.
